# Molecular Imaging in Tumor Angiogenesis and Relevant Drug Research

**DOI:** 10.1155/2011/370701

**Published:** 2011-07-25

**Authors:** Xibo Ma, Jie Tian, Xin Yang, Chenghu Qin

**Affiliations:** Intelligent Medical Research Center, Institute of Automation, Chinese Academy of Sciences, Beijing 100190, China

## Abstract

Molecular imaging, including fluorescence imaging (FMI), bioluminescence imaging (BLI), positron emission tomography (PET), single-photon emission-computed tomography (SPECT), and computed tomography (CT), has a pivotal role in the process of tumor and relevant drug research. CT, especially Micro-CT, can provide the anatomic information for a region of interest (ROI); PET and SPECT can provide functional information for the ROI. BLI and FMI can provide optical information for an ROI. Tumor angiogenesis and relevant drug development is a lengthy, high-risk, and costly process, in which a novel drug needs about 10–15 years of testing to obtain Federal Drug Association (FDA) approval. Molecular imaging can enhance the development process by understanding the tumor mechanisms and drug activity. In this paper, we focus on tumor angiogenesis, and we review the characteristics of molecular imaging modalities and their applications in tumor angiogenesis and relevant drug research.

## 1. Introduction

Drug development, especially antitumor drug development, is a relatively long process. Drug development contains five procedures, assessment of target expression, lead compound optimization, pre-Phase 1 studies, clinical Phase 1–3 studies, and Federal Drug Association (FDA) approval [1–3]. Angiogenesis is one of the fundamental characteristics during tumor progression and metastasis [4]. Detection of newborn tumor blood vessels and antiangiogenic drugs are being widely studied. Traditional biological techniques usually require euthanasia of experimental animals to acquire the correlative biological information of interest which make continuous detection impossible. 

In recent years, molecular imaging emerged with increasing popularity in monitoring the changes at a molecular level *in vivo* and in evaluating treatment efficacy much earlier and much more accurately [5]. In contrast with traditional biological technology, molecular imaging can provide a whole body readout in an intact system through a longitudinal study in the same animals, provided that more statistically relevant and more accurate results are presented in evaluating the efficacy of antitumor drugs [6–8]. Because of the characteristics of molecular imaging, it can certainly decrease the workload and speed up drug development. In this paper, we review the characteristics and principles of different molecular imaging modalities before describing how molecular imaging can be used in tumor angiogenesis detection and relevant drug development.

## 2. Characteristics and Principles of Molecular Imaging

**Figure 1:**
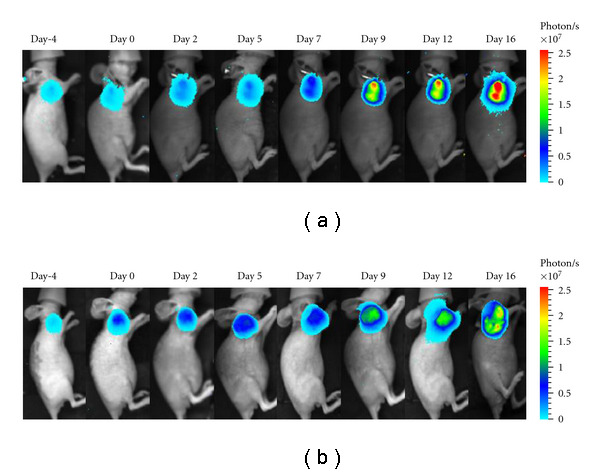
Serial bioluminescence images of the HCC-LM3-fLuc tumor-bearing nude mice that underwent saline (a) or cyclophosphamide (b) treatment (data from Ma et al. [6]).

Molecular imaging can monitor biological processes at cellular and molecular levels in intact living subjects. Optical molecular imaging, such as bioluminescence imaging (BLI) and fluorescence imaging, has the advantage of high sensitivity and low cost. BLI uses light produced by the enzymatic reaction of a luciferase enzyme and its substrate. Based on this imaging principle, split-protein strategies were developed to assess protein-protein interactions *in vivo* using BLI techniques [9–19]. By detecting the bioluminescence photons, we can characterize a variety of cellular and molecular processes, including protein interactions [20–23], protein degradation [24–26], and protease activity [27–29]. Otherwise, the firefly luciferase gene could be transferred into tumor cells and could be used to evaluate the tumor response to chemotherapeutic agents in living animals. In Figure 1 [6], we used BLI to evaluate the antitumor efficacy of cyclophosphamide in HCC-LM3-fLuc tumor-bearing nude mice.

After imaging acquisition, we could count the photons in a region of interest (ROI) to quantify the antitumor efficacy by using WINMI (windows-based molecular imaging), which was developed based on the medical imaging ToolKit (MITK [30]; Medical Image Processing and Analyzing group, Institute of Automation, Chinese Academy of Sciences, Beijing, China;  http://www.mitk.net/).

**Figure 2:**
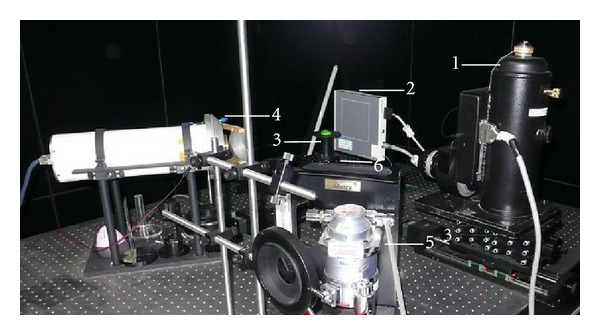
Our prototype BLT/Micro-CT dual modality imaging system. (1) CCD camera; (2) X-ray detector; (3) mouse holder; (4) X-ray tube; (5) anesthesia machine; (6) rotation stage.

The tumor volume was measured using a calendar in order to evaluate the antitumor efficacy of the drug. However, conventional measurement with a calendar usually introduced more induced errors. Through quantification of the light intensity, this BLI technique can provide more accurate information of tumor progression. However, this technique can only provide two-dimensional information on tumor which cannot fulfill the need for inner tumor detection and corresponding drug evaluation. Furthermore, when combined with computed tomography (CT), we have developed our prototype system to detect inner tumors in the mouse. The dual modality system (Micro-CT and BLI) is shown in Figure 2. Micro-CT can provide three-dimensional anatomic structure information to form the heterogeneous mouse which is proven to be more accurate in reconstructing the three-dimensional location information [31]. This technique provides an effective tool for inner tumor detection and evaluation of antitumor drugs. 

In fluorescence imaging (FMI), an external light with an appropriate wavelength was used to excite a target fluorescent molecule. Then, almost immediately, the target fluorescent molecules released a longer wavelength, lower-energy light for imaging. In contrast to BLI, the detection depth of FMI is greater than a few millimeters by using the near infrared fluorescence (NIRF) probe and dye [8]. After imaging acquisition and filtering of the background noises, the high-contrast images can be obtained. Using the same method with BLI, the light intensity can be counted to evaluate the tumor volume and antitumor drug efficacy. 

Positron emission tomography (PET) and single-photon emission-computed tomography (SPECT) are imaging modalities that are widely used in preclinical and clinical studies [32, 33]. They can be used to detect the molecular and cellular changes of diseases before the tumor is large enough to cause structural changes by injecting radiotracer labeled probes [34, 35] (such as ^18^F for PET, ^131^I for SPECT). These imaging modalities have been applied not only in rodents or larger animals, but also in clinical trials, such as for disease diagnosis [34] and monitored disease therapy [36]. Combined with morphological/anatomical imaging modalities (such as CT), PET/SPECT can be used to obtain three-dimensional tomography images and can provide dynamic functional information. Because of the decay characteristics of a radionuclide, time-decay calibration was often used to attain radionuclide uptake per weight for the comparison between different tissues. In contrast with BLI/FMI, PET/SPECT shares the advantage of having high sensitivity with unlimited depth penetration. However, they also share the limitations of high cost and limited spatial resolution. 

CT is widely used in diagnostic medicine as it enables doctors to provide safe, cross-sectional images with a high resolution to patients. It uses X-rays for forming tissue images, and the images are reconstructed from its projections. A projection means a line integral to the X-ray attenuation coefficients of the images in a given direction. In preclinical research, Micro-CT for small animal imaging has played a critical role in the evolution of molecular imaging [37]. The X-Ray data was reconstructed based on cone-beam geometry utilizing a Feldkamp-Davis-Kress (FDK) algorithm [38], which is an approximate algorithm, and the cone angle should be limited to a relatively small value to avoid cone-beam artifacts. Micro-CT can obtain high resolution anatomic information which can integrate with other modalities, such as nuclear and optical approaches. Since X-ray attenuation of soft tissues is different from that of bones, Micro-CT is very suitable for bone detection, such as bone growth, destruction, and changes in bone density. Despite lacking a targeted contrast agent, Micro-CT is not able to acquire molecular imaging directly, but it can indirectly reflect the changes of cells and molecular structures, such as imaging changes in vascular density [39]. For *in vivo* imaging, especially in a longitudinal study, the radiation dose should be considered, and much attention has been paid to CT imaging with a high resolution as well as a low-radiation dose.

## 3. Tumor Angiogenesis

Tumor angiogenesis is a process of proliferation of a network of blood vessels that penetrates into cancerous growths, supplying nutrients and oxygen and removing waste products. Tumor angiogenesis is activated by the signal molecules that are sent by cancerous tumor cells. These signals activate certain genes in the host tissue, make proteins, and form the network of new blood vessels surrounding the tumor. These signal molecules include, but are not limited to, growth factor receptors, tyrosine kinase receptors, integrins, and matrix metalloproteinases [40] (MMP). In this paper, we will focus on two of the most intensively studied angiogenesis-related molecular targets: vascular endothelial growth factor (VEGF) and VEGF receptors (VEGFR), and integrin *α*_*v*_*β*_3_. During tumor angiogenesis, these molecules can be used as indicators in molecular imaging. By targeting them, we can diagnose some tumors which express these molecules. Furthermore, we can evaluate the antitumor efficacy of drugs using the correlative probes. 

## 4. Imaging VEGF/VEGFR Expression

VEGF is a powerful mitogen in embryonic and somatic angiogenesis. It plays a pivotal role in both normal vascular tissue development and tumor neovascularization. Overexpression of VEGF is associated with tumor progression and poor prognosis in several tumors, including colorectal, gastric, and pancreatic carcinomas; angiosarcomas; breast, prostate, and lung cancers; malignant gliomas; melanoma. 

Through building a transgenic mouse of a VEGF promoter driven GFP reporter gene, Izzo's group proved fluorescence imaging is credible in detecting the VEGF [41]. Afterwards, Wang's group used BLI to detect VEGF in a transgenic mouse model [42]. Vascular endothelial growth factor A (VEGFR-1) and Flk-1/KDR (VEGFR-2) are key regulators for tumor angiogenesis and tumor growth. Chen's group monitored the antiangiogenic and antitumor efficacies of a vasculature-targeting fusion toxin (VEGF_121_/rGel) composed of the VEGF-A isoform VEGF_121_ linked with a G_4_S tether to recombinant plant toxin gelonin (rGel) in an orthotopic glioblastoma mouse model by use of noninvasive *in vivo* BLI, magnetic resonance imaging (MRI), and PET. Through this study, they proved multimodality imaging and therapy with VEGF_121_/rGel could provide an effective means to prospectively identify patients who will benefit from VEGF_121_/rGel therapy and then stratify, personalize, and monitor treatment to obtain optimal survival outcomes in future clinical applications [43]. 

As an effective imaging technique, PET has been applied in the clinical studies. With the development of Micro-PET scanners dedicated to small animal imaging studies, it can provide a similar *in vivo* imaging capability in mice, rats, monkeys, and humans [44, 45]. As an IgG1 monoclonal antibody that binds to human VEGF, VG76e was labeled with ^124^I for PET imaging of solid tumor xenografts in immune-deficient mice [46]. Chen's group [47] labeled VEGF_121_ with ^64^Cu for PET imaging of tumor angiogenesis and VEGFR expression. This study showed that ^64^Cu-DOTA-VEGF_121_ (~15% ID/g) had a rapid, specific, and prominent uptake in highly vascularized small U87MG tumors with high VEGFR-2 expression but lower and sporadic uptake in large U87MG tumors with low VEGFR-2 expression [47]. In a follow-up study, a VEGFR-2 specific fusion toxin VEGF_121_/rGel (composed of VEGF_121_ linked with a G_4_S tether to the recombinant plant toxin gelonin) was used to treat orthotopic glioblastoma in a mouse model [48]. In addition, recombinant human VEGF_121_ was labeled with ^111^In for SPECT imaging to identify ischemic tissue in a rabbit model, where unilateral hind-limb ischemia was created by femoral artery excision [49]. Furthermore, VEGF_121_ has also been labeled with ^99m^Tc through an “adapter/docking” strategy, and the tracer was tested in a 4T1 murine mammary carcinoma with tumor uptake of about 3% ID/g [50, 51].

## 5. Imaging *α*_*v*_*β*_3_ Expression

*α*_*v*_ *β*_3_ is a member of the integrin family which is a kind of adhesion molecule consisting of two noncovalently bound transmembrane subunits (*α* and *β*). In mammals, 18*α* (120–185 KD) and 9*β* (90–110 kd) subunits assemble into 24 kinds of different receptors. Integrin signaling pathways play a crucial role in tumor angiogenesis and metastasis. Tumor growth, angiogenesis, and metastasis can be inhibited by blocking integrin signaling. Integrin *α*_*v*_*β*_3_ is an important indicator in evaluating the antitumor efficacy of drugs to the *α*_*v*_*β*_3_ positive tumors. Some antibodies, peptides, peptidomimetics, and other antagonists have been proven to have great potential in the treatment of cancer. 

**Figure 3:**
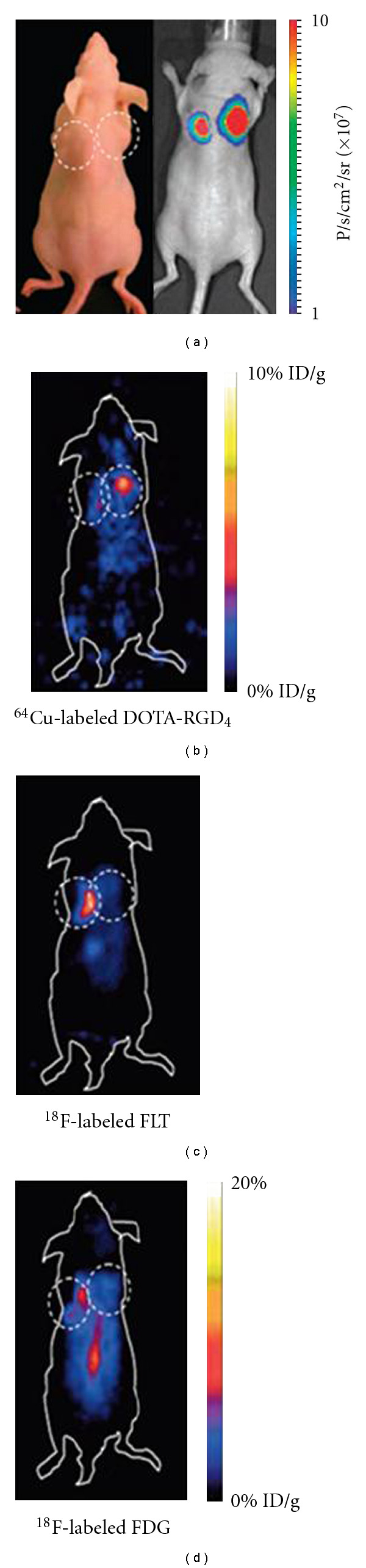
Micro-PET imaging of ^18^F-FDG, ^18^F-FLT, and ^64^Cu-DOTA-RGD4 on hES cell-derived teratoma. (a) Background imaging and bioluminescence imaging show teratoma formation in H9Hes-DF cells in the right shoulder and tumor formation in the control 2008 cell line in the left shoulder (7.3 ± 2.5 × 10^7^ versus 4.5 ± 1.6 × 10^7^ photon/s/cm^2^/sr, resp.); (b) specific and prominent uptake of ^64^Cu-DOTA-RGD4 was found in the vascularized teratoma but not in the control human ovarian carcinoma 2008 cell line with low integrin expression (*P* < 0.01); (c) hES cell-derived teratomas had a low uptake of ^18^F-FLT, whereas the 2008 cancer xenografts exhibited high ^18^F-FDG and ^18^F-FLT uptakes; (d) hES cell-derived teratomas had a low uptake of ^18^F-FDG, whereas the 2008 cancer xenografts and abdomen exhibited high ^18^F-FDG and ^18^F-FLT uptakes (data from Cao et al. [58]).

Integrin *α*_*v*_*β*_3_ is often expressed on some tumor cells in the brain, such as glioblastoma multiforme, and striatum neutral stem-cells-derived primary tumor. Via radiotracer labeling of some ligands or antibodies, the expression of integrin *α*_*v*_*β*_3_ can be imaged by Micro-PET or SPECT [52]. In Jia's research [53], a ^99m^Tc-Labeled cyclic RGDfK dimer has been studied to evaluate its application for SPECT imaging of glioma integrin *α*_*v*_*β*_3_ expression. They found that glioma tumors could be clearly visualized with all three radiotracers at 4 h after injection and 99mTc(SQ168)(tricine)(TPPTS) (*(SQ168) [2-[[[5-[carboonyl]-2-pyridinyl]-hydrazono]methyl] benz-enesulfonic acid]-Glu(cyclo{Lys-Arg-Gly-Asp-D-Phe})-cyclo{Lys-Arg-Gly-Asp-D-Phe}) had the best imaging quality among the three radiotracers [53] ([^99m^Tc(SQ168)(EDDA)], [^99m^Tc(SQ168)(tricine)(PDA)], and [^99m^Tc(SQ168)(tricine)(TPPTS)] (SQ168 =[2-[[[5-[carboonyl]-2-pyridinyl]-hydrazono]methyl]benzenesulfo-nic acid]-Glu (cyclo{Lys-Arg-Gly-Asp-D-Phe})-cyclo{Lys-Arg-Gly-Asp-D-Phe}; EDDA = ethylenediamine-*N*,*N *′-diacetic acid; PDA = 2,5-pyridinedicarboxylic acid; TPPTS = trisodium triphenylphosphine-3,3′,3′′-trisulfonate). ^99m^Tc-labeled RGD has also been used in gamma imaging of secondary tumors of transplanted human fetal striatal neutral stem-cells-derived primary tumor cells [54]. The results showed both PET and SPECT could be used in imaging tumorigenesis and metastasis of integrin *α*_*v*_*β*_3_ positive tumors. Some other radiotracers included ^18^F-[55, 56], ^68^Ga- [57], and ^64^Cu-[58] labeled RGD that were applied in imaging the integrin *α*_*v*_*β*_3_ expression using PET or SPECT. All of the radiotracers also validated the potential value in preclinical and clinical studies. In Cao's research [58], ^64^Cu-labeled RGD was used in imaging teratoma formation derived from hES cells by targeting *α*_*v*_*β*_3_ integrin in a nude mouse. Furthermore, by comparing it to BLI, ^64^Cu-DOTA-RGD4 proved to have the ability of noninvasively visualizing teratoma formation *in vivo*. Compared with the two other probes, ^18^F-labeled FLT and 18F-Labeled FDG, ^64^Cu-DOTA-RGD4 has a higher sensitivity and tumor to background contrast (data shown in Figure 3). ^ 90^Y-[59] and ^111^IN-[60] labeled monoclonal antibody abegrin had also been studied in imaging *α*_*v*_*β*_3_ expression of U87 glioblastoma multiforme.

**Figure 4:**
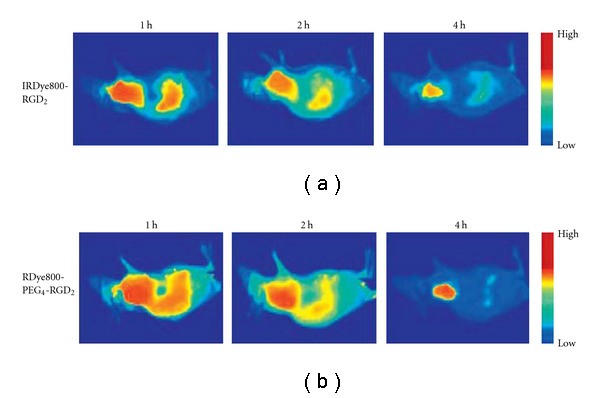
*In vivo* fluorescence imaging of U87MG tumor-bearing nude mice at 1, 2, and 4 hours after intravenous injection of 0.5 nmol IRDye800-RGD2 or IRDye800-PEG4-RGD2. Fluorescence signal from IRDye800 is pseudocolored (data from Liu et al. [61]).

 Otherwise, fluorescent dye-labeled E [PEG_4_-c(RGDfK)]_2_ had been developed to image integrin *α*_*v*_*β*_3_ expression by Liu and his coworkers [61]. By comparing the characterization of dye-labeled E [PEG_4_-c(RGDfK)]_2_ with dye-labeled RGD dimer without PEG_4_ linkers, Cy5.5-labeled E [PEG_4_-c(RGDfK)]_2_ showed higher tumor accumulation and tumor-to-background contrast (data shown in Figure 4). *In vivo* NIRF imaging with IRDye800 E [PEG_4_-c(RGDfK)]_2_ was confirmed to offer an easy, fast, and low-cost way to detect and semiquantify tumor integrin *α*_*v*_*β*_3_ expression in living subjects. 

Integrin *α*_*v*_*β*_3_ is one of the most extensively studied molecular targets involved in tumor angiogenesis [62–65]. Markiewicz's group had discovered a presumably inactive linear hexapeptide GRDSPK with near-IR carbocyanine molecular probe yielding Cyp-GRD which could be used to target integrin *α*_*v*_*β*_3_-positive tumors [66]. They later synthesized and evaluated a series of multimeric RGD compounds constructed on a dicarboxylic acid-containing near-IR fluorescent dye cypate for tumor targeting [67]. Furthermore, optimization of the spatial alignment and ligand reconstructed of the RGD moieties through careful molecular design may induce multivalent ligand-receptor interactions useful for *in vivo* tumor imaging and tumor-targeted therapy. As a humanized monoclonal antibody against human integrin *α*_*v*_*β*_3_ (picomolar binding affinity), abegrin (MEDI-522, also called Vitaxin; MedImmune, Inc., Gaithersburg, Md) is used in clinical trials for cancer therapy. Tice's group had conjugated abegrin with macrocyclic chelating agent DOTA and labeled it with ^64^Cu for PET imaging of a tumor xenograft [68]. Numerous reports on multimodality molecular imaging of integrin *α*_*v*_*β*_3_ showed that tumor angiogenesis imaging can participate in multiple stages of the drug development process, including target validation, lead compound optimization, compound screening, and clinical trials. 

## 6. Conclusion and Perspective

Molecular imaging has been widely applied in preclinical studies using some radiotracer probes, and PET and SPECT have been used in clinical diagnoses and antitumor evaluation of some drugs. However, optical molecular imaging especially BLI can only be used in preclinical studies. Many problems can be solved by applying molecular imaging in clinical research. 

BLI has more widely used applications in tumor detection and evaluation of pharmacodynamics, toxicity, and pharmacokinetics because of its noninvasive molecular and cellular level detection ability, high sensitivity, and low cost in comparison with other imaging technologies. However, BLI cannot present the accurate location and intensity of the inner bioluminescent sources such as in the bone, liver, or lungs. Bioluminescent tomography (BLT) shows its advantages in determining the bioluminescent source distribution inside a small animal or phantom. By utilizing CT information acquired by an X-ray detector, the three-dimensional location can be reconstructed using some BLT reconstruction methods such as the adaptive finite element method and Bayesian method These tomography imaging methods can be used in early detection of tumors [69] and assist in the diagnosis and evaluation of drug efficacy more accurately. Furthermore, the clinical application of BLI needs to develop some novel probes which can be used in humans. Developing a novel imaging probe is an expensive, time-consuming procedure. 

For PET/SPECT imaging, some radiotracers such as ^18^F, ^64^Cu, ^111^In, and ^68^Ga have been used in labeling ligands and antibodies to image their relative receptors. However, the binding ability between some radiotracers, their ligands, and antibodies is low. Some single-targeted probes have poor pharmacokinetic properties with fast clearance speed. Furthermore, with the development of molecular imaging, some new dual-modality or multimodality probes need to be developed to acquire more general, more accurate information of the disease. In recent years, significant advances have been made in developing novel probes for multimodality molecular imaging of tumor angiogenesis. Among all of the studies regarding multimodality probes, multireceptor-targeted imaging probes are most popular with some advantages. First of all, because some receptors may not be sufficiently expressed in all tumor cells during tumor progression and processes, with multireceptor targeting, more probes can accumulate in tumor cells and tissues. Secondly, the binding affinity of multireceptor-targeted probes can be relatively higher than in single receptor targeted probes. Thirdly, the clearance properties and excretion rates of dual- or multireceptor-targeted probes may be optimal compared to single receptor-targeted probes. In general, developing novel, high-affinity probes is necessary for molecular imaging.

In summary, although imaging to tumor angiogenetic and relevant drugs have been reported in previous work, tumor angiogenesis and relevant drug studies are subject to many problems, such that a good probe for imaging VEGF/VEGFR is limited which greatly hinders the development of imaging; imaging sensitivity needs to be improved which can help in determining whether or not to start and when to start VEGFR-targeted treatment. Accordingly, research on the following two aspects need to be strengthened in the future.

Based on the current probe, the development of a more-sensitive, less-toxic multimodal molecular imaging probe is necessary.

Based on the current imaging technique, the development of more accurate image processing algorithms is the key to VEGF/VEGFR and relevant drug studies.
